# A Latent‐factor MCACE Model for Multidimensional Outcomes and Treatment Noncompliance with Application to a Longitudinal Trial of Arthritis Health Journal

**DOI:** 10.1002/sim.70532

**Published:** 2026-04-26

**Authors:** Lulu Guo, Yi Qian, Diane Lacaille, Hui Xie

**Affiliations:** ^1^ Department of Data Science Dana‐Farber Cancer Institute Boston Massachusetts USA; ^2^ Sauder School of Business University of British Columbia British Columbia Canada; ^3^ Department of Medicine University of British Columbia British Columbia Canada; ^4^ Faculty of Health Sciences Simon Fraser University British Columbia Canada; ^5^ Arthritis Research Canada Vancouver British Columbia Canada

**Keywords:** causal inference, factor analysis, mixed‐effects model, potential outcome model, principal stratification, treatment effects estimation

## Abstract

Real‐world randomized controlled trials (RCTs) evaluating multifaceted interventions often employ multiple study outcomes to measure treatment effects on a small set of underlying constructs. Motivated by a longitudinal RCT evaluating a behavioural intervention, the Arthritis Health Journal (AHJ), we propose a latent‐factor multivariate complier average causal effects (MCACE) model for multidimensional longitudinal outcomes with principal strata of compliance types for parsimonious estimation of intervention effects in RCTs with treatment noncompliance. Within each compliance type, a factor regression model relates multiple outcomes to latent constructs, which follow hierarchical regression models. Under the model, high dimensional outcomes are reduced to low dimensional latent factors. This dimension reduction leads to a parsimonious and efficient test of overall CACEs on multiple outcomes, mitigating the multiple testing issues associated with multidimensional outcomes and weak instrumental variable problems associated with low compliance rates. Simulation studies demonstrate that the latent‐factor MCACE model outperforms univariate CACE analysis in terms of both statistical power and Type I error control. The application to the AHJ study selects two underlying factors (self‐efficacy and interaction with health care providers). Significant and beneficial treatment effects are detected on both factors. Overall, our analysis directly answers the main scientific questions posed by the RCT and yields novel findings not discovered previously.

## 1 Introduction

Randomized controlled trials (RCTs) are the preferred study design to assess intervention effects for healthcare policy decision makings. However, individuals randomized to an intervention group often do not comply with the assigned treatment, particularly trials evaluating complex interventions such as behavioural interventions. With treatment noncompliance, standard intention‐to‐treat (ITT) analysis typically provides conservative estimates of intervention efficacy [1]. To overcome this limitation, the complier average causal effect (CACE)—the principal causal effect (PCE) within the stratum of compliers—has been developed to estimate the intervention efficacy for the subpopulation who would comply regardless of assigned treatment [2, 3, 4]. CACE has been considered as patient‐oriented intervention effect of interest under treatment noncompliance [5].

Furthermore, real‐world RCTs evaluating multifaceted interventions often employ multiple study outcomes (also known as endpoints) to measure a limited set of underlying latent constructs, such as psychological traits, mental health status, quality of life, self‐efficacy, knowledge, and attitudes. Multifaceted interventions contain multiple components designed to impact a set of underlying constructs (e.g., self‐efficacy in disease management and effectiveness in shared decision‐making), each of which is measured by several study outcomes. Frequently, these multiple study outcomes are collected longitudinally on study participants, yielding multidimensional longitudinal outcomes. Evaluating CACEs for each outcome separately can face the challenges associated with multiple testing, a significant loss of statistical power, an inability to directly answer key scientific questions regarding treatment efficacy on the underlying constructs, and difficulty in interpreting potentially conflicting results among individual outcomes. To overcome these limitations, this work develops a latent‐factor model for parsimonious CACE estimation of intervention effects in RCTs with multidimensional longitudinal outcomes and treatment noncompliance.

The application motivating this work is the Arthritis Health Journal (AHJ) study, an RCT comparing the AHJ (intervention group) with the usual care (control group) in managing rheumatoid arthritis (RA) [6, 7]. As there has been no cure for RA, to achieve optimal health outcomes, people with RA must engage in efficient self‐management and effective collaboration with their healthcare providers [8, 9]. AHJ is a patient‐centered online tool designed to improve self‐efficacy in disease management and shared decision‐making for patients living with rheumatoid arthritis. In helping RA patients better monitor their disease activity and collaborate with their healthcare providers, this tool aims to facilitate the treat‐to‐target approach by providing early signs when the disease is not controlled. In the RCT, RA patients randomized to the AHJ were provided with online access to the AHJ after randomization and were asked to use it for 6 months, while those randomized to the control group did not have access to the AHJ during the 6‐month period.

The primary objective of this study is to evaluate the treatment efficacy of the AHJ tool on patients' self‐efficacy in disease management and their effectiveness in shared decision‐making. To effectively capture these complex underlying constructs, the AHJ study employed a total of six study endpoints (effective consumer 17 scale, manage symptom scale, manage disease in general scale, partners in health scale, communicate with physician scale, and satisfaction with medical care) measured using self‐reported questionnaires administered at baseline, three months and six months after baseline. In addition to study endpoints, the baseline questionnaires collected demographic and disease information, and the follow‐up questionnaires administered at 3 and 6 months also evaluated the frequency of using the AHJ online tool. As with several other such trials, treatment noncompliance occurred in the AHJ study, and a significant number of study participants randomized to the AHJ did not use or only rarely used the tool during the study period. A secondary objective of this study is to investigate predictors of compliance behaviour of study participants.

In the presence of such treatment noncompliance, traditional intention‐to‐treat or as‐treated analyses can yield biased estimates of the intervention effects of interest. To achieve our primary objective, an attractive alternative is to evaluate the effect of an intervention on the outcomes, adjusting properly for the treatment noncompliance using the principal stratification (PS) approach. In the context of treatment noncompliance, proper analysis adjusts for the principal strata corresponding to compliance types formed by the joint potential compliance behaviours under both control and intervention. As such defined, the values of principal strata are unaffected by the treatment assignment and behave like a baseline categorical variable. Thus, one can define the causal effects within each subpopulation determined by the compliance types. An intervention effect estimand of great interest is the CACE, the intervention effects in the subpopulation of compliers who would comply regardless of treatment assigned [2, 4].

The estimation of CACEs for AHJ requires handling multidimensional longitudinal outcomes, which raises several statistical issues. One such issue is the presence of multiple endpoints that measure different aspects of a small set of underlying constructs. CACE estimation requires appropriate statistical methods that can jointly consider all correlated endpoints and combine information from them for efficient and interpretable treatment effect estimation. Statistical methods permitting parsimonious testing of CACEs and mitigating the multiple testing issues in the presence of multiple endpoints are thus a desideratum. Finally, these methods must account for the longitudinal correlations among repeated measures and cross‐sectional correlations among multiple endpoints.

One approach is to estimate CACE separately for each individual endpoint. While this approach is straightforward in its application, if the correlations across multiple outcomes are not considered, the method is inefficient and can significantly diminish the study's power to detect intervention effects. Besides the multiple testing issue associated with analyzing these endpoints individually, the results can be difficult to interpret because of lacking direct connection with the underlying constructs of main interest and potentially conflicting results among individual endpoints. To improve the efficiency of CACE estimation in the presence of treatment noncompliance, most works focus on modelling two outcomes jointly in a cross‐sectional setting. Jo and Muthén [10] employed a secondary outcome to increase the precision of identifying compliance class and the power to detect intervention effects on the primary outcome. Mattei et al. [11] proposed a Bayesian approach to exploit bivariate outcomes to sharpen inferences for weakly identified models within principal strata in the cross‐sectional setting. Mealli and Pacini [12] demonstrated that a secondary outcome helps tighten the nonparametric bounds of CACE. An exception to these studies is that of Guo et al. [7] who considered the CACE estimation for multidimensional longitudinal outcomes in RCTs with treatment noncompliance and showed that jointly modeling of multiple study endpoints significantly improves the precision of estimating CACE and the power to detect CACE for individual endpoints. However, none of these methods are designed to exploit the underlying constructs targeted in RCTs of multifaceted interventions, including the AHJ study. As such, these methods can suffer from multiple testing issues in the presence of multiple endpoints and yield results that are less conducive to interpretation.

In this paper, we introduce a latent‐factor multivariate CACE (MCACE) model that exploits underlying constructs for parsimonious CACE estimation in RCTs with multidimensional longitudinal outcomes and treatment noncompliance. Within each (potentially unobserved) compliance type, a latent‐factor hierarchical regression model is used to connect longitudinally measured multiple endpoints with latent factors representing underlying constructs. Separate linear mixed‐effects models are then used to model these latent factors and the principal causal effects on them. Compared with individualized analysis of the causal effects of multiple endpoints, latent factors can capture the correlations across multiple outcomes. Inference based on latent factors uses information from all sources more efficiently than inference that uses data separately from each individual endpoint, which helps address the weak instrumental variable problems caused by low compliance rates. Unlike alternative joint CACE estimation approaches [7, 10, 11, 12], our proposed approach exploits underlying constructs, thereby increasing the results' interpretability and mitigating the multiple testing issues arising with multiple endpoints. Unlike data reduction techniques using pre‐specified functions of individual endpoints (e.g., sum or weighted average) or variable reduction methods such as principal components analysis, our approach derives the underlying constructs and factor loading using all data on multidimensional outcomes at all time points, yielding results that have greater interpretability and efficiency.

We apply the proposed approach to evaluate the AHJ online tool's treatment efficacy. Model comparisons and substantive considerations select two underlying constructs (patients' self‐efficacy and interaction with health care providers), permitting more parsimonious and powerful tests of CACEs on the low‐dimensional latent constructs than CACE analysis applied separately to each of the six outcomes. In particular, using the proposed model, we can detect significant and beneficial CACEs of AHJ, adjusting for multiple testing issues, on both latent constructs that are scientifically relevant. The findings differ significantly from those achieved using the alternative joint CACE modeling approach that jointly models all six study endpoints directly without considering the underlying constructs. Specifically, in the joint CACE model proposed in Guo et al. [7], after rejecting the null hypothesis of no CACE for all six endpoints using a global test, they had to examine the CACEs for the six endpoints separately to determine the location (i.e., which endpoint?) and direction (i.e., beneficial or harmful?) of intervention effects. Statistically significant CACEs were found in only two of the six study endpoints after multiple testing adjustments. In comparison, the latent‐factor MCACE method proposed in this work yields a more efficient and clearer interpretation of the RCT data.

Next, we describe the methodology in Section 2. Section 3 describes the simulation studies, which demonstrate the proposed model's performance and advantages. We then apply the method to the AHJ data in Section 4. Finally, a discussion will follow in Section 5.

## 2 Methodology

### 2.1 Notation and Assumptions

Let $A_{i}$ denote the $i^{th}$ subject's group assignment, i=1,…,N. Participants are randomly assigned to either the intervention group ($A_{i}=1$) or the control group ($A_{i}=0$). Let $D_{i}(A_{i})$ indicate the receipt of the treatment if the $i^{th}$ individual was assigned to group $A_{i}$. In the AHJ study, we define $D_{i}(A_{i}=1)=1$ when subject i used the tool at least once per month on average within a six‐month period after being assigned to the intervention group and define $D_{i}(A_{i}=1)=0$ if otherwise [7]. Let A and D denote N‐dimensional vectors of $A_{i}$ and $D_{i}$, respectively. Two types of potential outcomes can be defined. $D_{i}(A)$ is the potential treatment received by subject i when subjects are randomized to A. Assuming that K endpoints were observed over time for each individual in the study, $Y_{ijk}(A,D)$ is the potential outcome for the $k^{th}$ endpoint collected at the $j^{th}$ time point for individual i under treatment assignment A and treatment receipt D, where j=0,1,…,J and k=1,…,K. In the AHJ study, j={0,1,2} for the baseline, third month, and sixth month, respectively, and K=6 for the six endpoints collected every three months for each participant. Let $Y_{i}(A,D)$ denote the vector of K∗(J+1) potential outcomes for subject i under treatment assignment A and treatment receipt D.

Table 1 summarizes the key assumptions made for the identification of the latent‐factor MCACE model in the study. Below, we first discuss Assumptions 1 to 4. These remaining assumptions (Assumptions 5 to 8) are related to latent factors and will be described later in the development of the latent‐factor MCACE model.

**TABLE 1:** Assumptions for the identification of latent‐factor MCACE model.

Assumptions	Statements
1: Stable unit treatment value assumption	No interference and no multiple versions of treatment.
2: Random assignment	Assignments are independent of potential outcomes given all observed baseline variables.
3: No access to the treatment in the control group	Rule out defiers and always‐takers.
4: Exclusion restriction	Y(A,D)=Y(D)
5: Conditional independence	Potential outcomes are conditionally independent given latent factors $U_{\mathrm{mij}}^{a}$.
6: Stability of factor loading matrix Λ	Λ is constant over time and across different compliance patterns.
7: Rotation restriction	Restrictions are imposed on matrix Λ to fix its rotation under both confirmatory and exploratory analyses.
8: Location and scale normalization	$x_{\mathrm{qij}}^{a}$ and $z_{\mathrm{qij}}$ in the level‐2 model do not include intercepts; $\epsilon_{qmij}^{a}∼N(0,1)$.

Assumption 1 Stable Unit Treatment Value Assumption (SUTVA [13, 14, 15]).

The SUTVA assumption assumes no interference and no multiple versions of treatment. The former one implies that an individual's potential outcomes are not influenced by others' treatment assignments, and the latter assumes that each individual receives exactly the same version of the treatment. The SUTVA assumption helps define the unit level causal effect and allows us to simplify $Y_{i}(A,D)$ and $D_{i}(A)$ as $Y_{i}(A_{i},D_{i})$ and $D_{i}(A_{i})$. SUTVA is satisfied in AHJ study because participants are independently provided with access to exactly the same AHJ tool.

Assumption 2 Random assignment.

This assumption implies that the assignment $A_{i}$ is independent of potential outcomes $Y_{i}(A_{i},D_{i})$ and $D_{i}(A_{i})$ given all observed baseline variables. Randomized controlled trials satisfy the randomization assumption by randomly assigning participants to the intervention and control groups.

Assumption 3 Patients in the control group do not have access to the treatment.

In the AHJ study, participants assigned to the control group had no access to the AHJ online tool during the first six‐month period, satisfying Assumption 3. Given that $D_{i}(A_{i})$ is a binary variable, the combination of potential outcomes $\left(D_{i}(1),D_{i}(0)\right)$ defines four possible compliance patterns: compliers (1,0), never‐takers (0,0), always‐takers (1,1) and defiers (0,1). Under Assumption 3, $D_{i}(0)=0$ for all individuals, ruling out defiers and always‐takers. Compliers (1,0) and never‐takers (0,0) can be distinguished in the treatment group because $D_{i}(1)$ is observable in the treatment arm. However, compliers and never‐takers can not be distinguished in the controlled group since $D_{i}(1)$ is not observable for the individuals assigned to the control group. Let $C_{i}$ be the compliance type of the participant i, $C_{i}\in \{c,n\}$ where c denotes compliers and n denotes never‐takers. $C_{i}$ is observable in the treatment group and unknown in the control group.

In other RCTs in which controls can take the treatment, Assumption 3 does not hold, and the monotonicity assumption is often invoked to rule out certain compliance types. For example, Hirano et al. [16] employs the monotonicity assumption ($D_{i}(1)\geq D_{i}(0)$) to rule out defiers for model identification and makes inference based on the population consisting of compliers, never‐takers and always‐takers. Our proposed method is general and can be extended to these situations when Assumption 3 is relaxed.

Assumption 4 Exclusion restriction [2, 3].

This assumption implies $Y(A,D)=Y(A^{\prime },D)$ $\forall A,A^{\prime }$ and ∀D, which implies Y(A,D)=Y(D)—that is, no difference in potential outcomes under treatment and control assignments for never‐takers. In practice, the plausibility of the exclusion restriction assumption can depend on the definition of compliance. In our AHJ study, we will conduct a sensitivity analysis to evaluate the likely dependence of CACE analysis on the assumption.

### 2.2 Models

We proposed a latent‐factor model with principal strata for partially observed compliance types under the potential outcome framework. In the AHJ study, follow‐up questionnaires employ multiple endpoints to measure the underlying constructs (factors): patients' self‐efficacy and satisfaction with health professional care and communication. These latent factors are of primary interest in the study but cannot be directly observed. Instead, multiple endpoints are used to measure these latent factors, which capture their interdependence. It is thus relevant to directly estimate the causal effect of the intervention on the latent factors.

**FIGURE 1:**
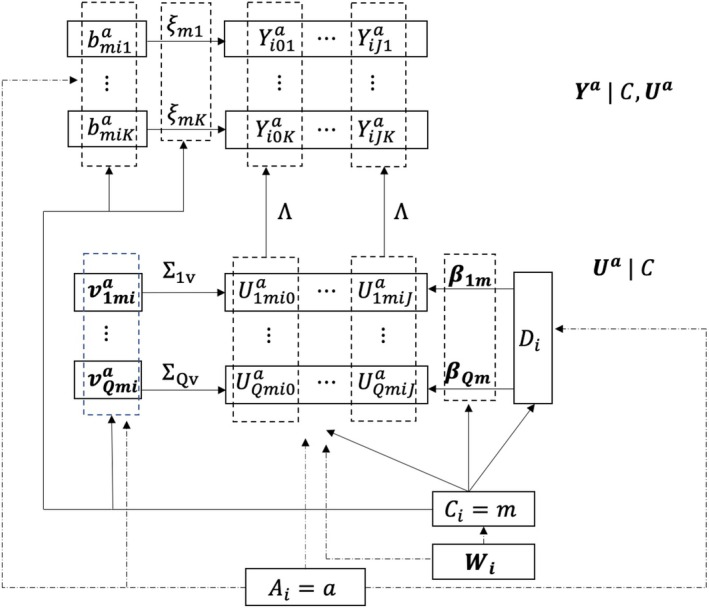
The structure of the latent‐factor MCACE model with principal strata for latent compliance types.

To achieve the above goal, we introduce the latent factor $U_{i}^{a}$ when modelling the joint distribution of potential treatment received $\left(D_{i}(0),D_{i}(1)\right)$ and potential outcomes of multiple endpoints $\left(Y_{i}^{0},Y_{i}^{1}\right)$, where $Y_{i}^{a}$ denotes the potential outcome $Y_{i}(D_{i}(A_{i}=a)),a=0,1$. Specifically, we model $(Y_{i}^{0},Y_{i}^{1})\;|\;C_{i},U_{i}^{a}$ in which the compliance type $C_{i}$ has one‐to‐one correspondence to $\left(D_{i}(0),D_{i}(1)\right)$, and the latent factor $U_{i}^{a}$ captures the interdependence of multiple endpoints induced by sharing a common set of latent factors in $U_{i}^{a}$ within the compliance type $C_{i}$. We then model the conditional distribution $U_{i}^{a}\;|\;C_{i}$, which captures the CACEs on the latent factors in $U_{i}^{a}$. Finally, the compliance behavior $C_{i}$ is modelled by using a logistic regression model. The diagram in Figure 1 outlines the model structure with modelling details described below.

#### 2.2.1 Model for $Y_{i}^{a}\;|\;C_{i},U_{i}^{a}$

Let $Y_{\mathrm{ij}}^{a}={\left(Y_{ij1}^{a},\ldots ,Y_{ijK}^{a}\right)}^{T}$. The level‐1 part of our multi‐level model specifies the relationship between $Y_{\mathrm{ij}}^{a}$ and the latent factors $U_{\mathrm{mij}}^{a}$ given the compliance type $C_{i}=m$ as

(1) $$\left.Y_{\mathrm{ij}}^{a}\;\right|\;\left(C_{i}=m,U_{\mathrm{mij}}^{a},b_{\mathrm{mi}}^{a}\right)=\lambda_{m0}+\Lambda U_{\mathrm{mij}}^{a}+b_{\mathrm{mi}}^{a}+e_{\mathrm{mij}}^{a},$$

where m∈{c,n} denotes the unique value of compliance type; $U_{\mathrm{mij}}^{a}={\left(U_{1mij}^{a},\ldots ,U_{Qmij}^{a}\right)}^{T}$ are Q (Q≪K) latent factors for subject i at time j with group assignment a and compliance type m; Λ is a K by Q matrix of regression coefficients with $\lambda_{kq}$ at the $k^{th}$ row and $q^{th}$ column, which does not change over time and across different compliance patterns; $\lambda_{m0}={\left(\lambda_{m01},\ldots ,\lambda_{m0K}\right)}^{T}$ is the average baseline measurements for control group subjects under the compliance type m; $b_{\mathrm{mi}}^{a}={\left(b_{mi1}^{a},\ldots ,b_{miK}^{a}\right)}^{T}$ with $b_{mik}^{a}$ representing the $k^{th}$ outcome's random intercept. In the above level‐1 model, the latent factors $U_{\mathrm{mij}}^{a}={\left(U_{1mij}^{a},\ldots ,U_{Qmij}^{a}\right)}^{T}$ capture the variability and interdependence among the K responses at each time point j. For the $k^{th}$ endpoint, $b_{mik}^{a}$ captures the correlation across longitudinal measurements of $y_{ijk}^{a}$ over time. We assume that $b_{mik}^{a}$ follows a normal distribution with mean 0 and variance $\xi_{mk}$ and $b_{mik}^{a}\;⊥\;b_{mih}^{a}$, k≠h. Finally, the error term $e_{\mathrm{mij}}^{a}={\left(e_{mij1}^{a},\ldots ,e_{mijK}^{a}\right)}^{T}$ with $e_{mijk}^{a}$ is distributed independently as $N(0,\tau_{mk}^{2})$, $e_{mijk}^{a}\;⊥\;e_{mijh}^{a}$ for k≠h. We further assume $e_{mijk}^{0}\;⊥\;e_{mijk}^{1}$, $b_{mik}^{0}\;⊥\;b_{mik}^{1}$ and $e_{mijk}^{a}\;⊥\;b_{mik}^{a}$. This implies correlations over time and across different outcomes are captured by random effects and latent factors.

The level‐1 model makes the following key assumptions for model identification. First, the potential outcomes are conditionally independent given the latent factors $U_{\mathrm{mij}}^{a}$ (Assumption 5 in Table 1), which means that $b_{mik}^{a}\;(k=1,\ldots ,K)$ are independent. At time j, the cross‐sectional correlations among the potential outcomes $y_{ijk}^{a}\;(k=1,\ldots ,K)$ are induced by the common latent factors $U_{\mathrm{mij}}^{a}$. An insufficient number of factors may fail to adequately capture the correlations among the potential outcomes, leading to underfitting and a loss of important information. Conversely, including too many factors can result in overfitting, where the model captures noise rather than meaningful structure, thereby reducing generalizability, interpretability, and parsimony. To address this trade‐off, it is important to estimate and compare models with different numbers of factors. Examining the stability of results across these models provides a means of assessing sensitivity to factor dimensionality. Model selection can be guided by a combination of statistical criteria with substantive considerations regarding the interpretability of the factors. Ultimately, the choice of the number of factors should balance statistical fit with theoretical coherence, ensuring that the selected model both captures the observed correlations and yields factors that are substantively meaningful in the context of the study.

Second, the correlation between the potential outcomes and latent factors, Λ, remains the same over time and across different compliance patterns (Assumption 6 in Table 1), which is required for model identifiability and factor interpretability. Since the latent factors, $U_{mij}^{a}$, vary across time points and compliance types, allowing the factor loading matrix Λ in Equation (1) to also vary by time or principal strata can introduce identifiability issues. Furthermore, without Assumption 6, latent factors can also encounter interpretability issues. Because the meanings of latent factors are determined by the pattern of loadings of observed outcomes on each latent factor, this condition ensures that the interpretation of the factors is consistent over time and across compliance types. This assumption is analogous to the requirement in longitudinal studies that the definitions of outcome measures should remain consistent over time. Therefore, Assumption 6 ensures identifiability by requiring that the potential outcomes measure the same underlying latent constructs across time and principal strata, by preserving a consistent interpretation of the latent factors. Exploring alternative models that do not include this condition, and assessing their practical utility, is beyond the scope of this work.

Third, we need to enforce some restrictions to fix the rotation of factor loading matrix Λ (Assumption 7 in Table 1). For any orthogonal matrix T that satisfies $TT^{\prime }=T^{\prime }T=I$, $\Lambda U_{\mathrm{mij}}^{a}=\Lambda TT^{\prime }U_{\mathrm{mij}}^{a}=\Lambda^{*}U_{\mathrm{mij}}^{a*}$, where $\Lambda^{*}=\Lambda T$ and $U_{\mathrm{mij}}^{a*}=T^{\prime }U_{\mathrm{mij}}^{a}$. Since there are infinite possible orthogonal matrices, Λ can be rotated to $\Lambda^{*}$ in infinite ways. Rotation restriction is a standard practice of imposing additional constraints on factor loadings to resolve the indeterminacy of the factor model [17, 18]. It does not change the statistical fit, but makes the solution unique and interpretable. In confirmatory analysis, the structure of matrix Λ is specified based on the scientific relationships between potential outcomes and latent factors, which imposes sufficient constrains to fix the rotation of the matrix Λ. In exploratory analysis, we do not make any assumptions about the latent structure of potential outcomes except that we set $\lambda_{kq}=0$, q>k [19].

#### 2.2.2 Model for $U_{i}^{a}\;|\;C_{i}$

In the level‐2 model, we assume a linear mixed‐effects model to study the longitudinal latent factors $U_{qmij}^{a}$, q=1,…,Q, for individuals with compliance type m,

(2) $$\left.U_{qmij}^{a}\;\right|\;\left(C_{i}=m,v_{\mathrm{qmi}}^{a}\right)=x_{\mathrm{qij}}^{a}\beta_{\mathrm{qm}}+z_{\mathrm{qij}}v_{\mathrm{qmi}}^{a}+\epsilon_{qmij}^{a},$$

where $x_{\mathrm{qij}}^{a}$ and $z_{\mathrm{qij}}$ are vectors of covariates for fixed effects and random effects for the $q^{th}$ latent factor respectively. The vector of covariates ($x_{\mathrm{qij}}^{a}$) can include the receipt of the treatment, time trends, their interactions, and baseline characteristics (demographic information, disease severity, etc.), while $z_{\mathrm{qij}}$ is a subset of $x_{\mathrm{qij}}^{a}$. To minimize the risk of misspecifying the trajectories of the latent factors, one may adopt flexible specifications such as visit‐specific dummy variables or nonlinear growth models. In the AHJ study, which includes three visits at 0, 3, and 6 months, we employ a quadratic time trend model. This specification is sufficiently flexible and functions as a saturated model for the given time points, thereby minimizing the likelihood of trajectory misspecification. Within the latent principal stratum ($C_{i}=m$), the receipt of treatment is either deterministic (for never takers) or randomized (for compliers) [20]. This means that conditioning on the principal stratum ($C_{i}=m$) alone, even if $x_{\mathrm{qij}}^{a}$ contains no baseline covariates, is sufficient to ensure the uncorrelatedness between the treatment receipt and the error term $\epsilon_{qmij}^{a}$ as well as the random effect $v_{qmi}^{a}$ in Equation (2), permitting CACE estimation using standard regression methods if $C_{i}$ is fully observed. The challenge is that principal stratum $C_{i}$ is unobserved for individuals randomized to the control group. Although compliers are observed in the treatment group ($D_{i}(A_{i}=1)=1$), these compliers alone do not permit CACE estimation. Furthermore, comparison of compliers and noncompliers in the treatment group typically yields biased treatment effect estimation since they come from different principal strata and are thus not comparable given the self‐selection nature of compliance. In the level‐2 model, $\beta_{\mathrm{qm}}$ and $v_{\mathrm{qmi}}^{a}$ are vectors of fixed‐effect parameters and random‐effect parameters for the $q^{th}$ latent factor, respectively. The random effects $v_{\mathrm{qmi}}^{a}$ are used to model the correlation of repeated measurements of the $q^{th}$ latent factor $U_{qmij}^{a}$. We assume $v_{\mathrm{qmi}}^{a}∼N(0,\sum_{qv})$ and $v_{\mathrm{qmi}}^{a}\;⊥\;v_{\mathrm{hmi}}^{a},q\neq h$. Furthermore, $\epsilon_{qmij}^{1}\;⊥\;\epsilon_{qmij}^{0}$, $v_{\mathrm{qmi}}^{1}\;⊥\;v_{\mathrm{qmi}}^{0}$ and $\epsilon_{qmij}^{a}\;⊥\;v_{\mathrm{qmi}}^{a}$.

To ensure model identifiability, we make the following assumption (Assumption 8 in Table 1). Because the latent factors are unobserved, their location (mean) and scale (variance) cannot be identified from the data [17]. To make the factor model estimable, we impose normalization restrictions, which do not change the statistical fit, but make the solution unique and interpretable. Since the level‐1 part of our multi‐level model already includes intercepts ($\lambda_{m0}={\left(\lambda_{m01},\ldots ,\lambda_{m0K}\right)}^{T}$) and individual‐specific random intercepts ($b_{\mathrm{mi}}^{a}={\left(b_{mi1}^{a},\ldots ,b_{miK}^{a}\right)}^{T}$), neither $x_{\mathrm{qij}}^{a}$ nor $z_{\mathrm{qij}}$ includes intercepts so that the model can be identified. Similarly, for the sake of identifiability, the level‐1 model (Equation (1)) does not include covariates due to the incorporation of covariates in the level‐2 model (Equation (2)). Furthermore, we also assume $\epsilon_{qmij}^{a}\overset{iid}{∼}N(0,1)$ which fixes the scale of the latent factor $U_{qmij}^{a}$ to ensure the model identification.

**Principal causal effects in longitudinal data.** We are interested in the principal causal effects (PCEs) on the latent factors $U_{qmij}^{a}(q=1,\ldots ,Q)$ because these latent factors correspond to the underlying constructs of interest. PCEs are defined as the ITT effects of the treatment for subpopulations determined by compliance behavior. In longitudinal studies, the PCEs can change over time. To account for potentially dynamic PCEs, we evaluate time‐specific PCEs and define the PCE at time point j on the $q^{th}$ latent factor $U_{qmij}^{a}$ within the compliance pattern m as

(3) $$\begin{array}{l} {\text{PCE}}_{qmj}=𝔼\left(U_{qmij}^{1}-U_{qmij}^{0}|C_{i}=m\right)=\left(x_{\mathrm{qij}}^{1}-x_{\mathrm{qij}}^{0}\right)\beta_{\mathrm{qm}}, \end{array}$$

where m=c (complier) or =n (never‐taker). To assess the causal effect of assignment $A_{i}$ on latent factors among compliers at time point j, we consider the CACE, which is the PCE for compliers as below

(4) $$\begin{array}{ll} {\text{CACE}}_{qj} & ={\text{PCE}}_{qcj}=𝔼\left(U_{qcij}^{1}-U_{qcij}^{0}|C_{i}=c\right)\\ & =\left(x_{\mathrm{qij}}^{1}-x_{\mathrm{qij}}^{0}\right)\beta_{\mathrm{qc}}.\; \end{array}$$

The random effects $v_{\mathrm{qmi}}^{a}$ and the error term $\epsilon_{qmij}^{a}$ in Equation (2) vanish in the PCE and CACE formula (Equations (3) and (4)) because the expectations of these terms equal 0 within the principal stratum $C_{i}$.

Equation (4) shows that ${\text{CACE}}_{qj}$ can be obtained by comparing the expectation of $q^{th}$ latent factor $U_{qcij}^{1}$ for compliers in the treatment group with the expectation of $q^{th}$ latent factor $U_{qcij}^{0}$ for compliers in the control group. We also note here that treatment assignment $A_{i}$ does not influence potential outcomes $Y_{i}(D)$ directly under Assumption 4. Given that latent factor $U_{i}^{a}$ captures the characteristics of potential outcomes $Y_{i}(D)$, treatment assignment $A_{i}$ does not influence latent factor $U_{i}^{a}$ directly either. Consequently, the PCEs for never‐takers on the latent factors are zeros since the ITT effect for never‐takers compares potential outcomes under the control condition regardless of treatment assignment.

**Combining the level‐1 and level‐2 models.** Using the matrix notation, models (1) and (2) can be succinctly written as

(5) $$\begin{array}{ll} \left.Y_{i}^{a}\;\right|\;\left(C_{i}=m,U_{\mathrm{mi}}^{a}\right) & =\lambda_{m0}⊗1_{J+1}+\left(\Lambda ⊗I_{J+1}\right)U_{\mathrm{mi}}^{a}\\ & \;+b_{\mathrm{mi}}^{a}⊗1_{J+1}+e_{\mathrm{mi}}^{a},\\ \;U_{\mathrm{mi}}^{a} & =X_{i,a}\beta_{m}+Z_{i}v_{\mathrm{mi}}^{a}+\epsilon_{\mathrm{mi}}^{a}, \end{array}$$

where $Y_{i}^{a}=\{Y_{ijk}^{a}:j=0,\ldots ,J;k=1,\ldots ,K\}$, $e_{\mathrm{mi}}^{a}=\{e_{mijk}^{a}:j=0,\ldots ,J;k=1,\ldots ,K\}$, $U_{\mathrm{mi}}^{a}=\{U_{qmij}^{a}:q=1,\ldots ,Q;j=0,\ldots ,J\}$, $\beta_{m}=\{\beta_{qmpr}:p=0,\ldots ,P;r=0,\ldots ,R\}$, where P and R depend on the forms of model (2). $v_{\mathrm{mi}}^{a}={\left({v_{1\mathrm{mi}}^{a}}^{T},\ldots ,{v_{\mathrm{Qmi}}^{a}}^{T}\right)}^{T}$. $X_{i,a}$ and $Z_{i}$ are design matrices for fixed effects and random effects in the model for $U_{\mathrm{mi}}^{a}$. $\epsilon_{\mathrm{mi}}^{a}=\{\epsilon_{qmij}^{a}:q=1,\ldots ,Q;j=0,\ldots ,J\}$.

After combining the above level‐1 model and level‐2 model, an overall model for the potential outcomes for individual i with compliance type m can be obtained as

$$\begin{array}{ll} \left.Y_{i}^{a}\;\right|\;\left(C_{i}=m\right) & =\lambda_{m0}⊗1_{J+1}+\left(\Lambda ⊗I_{J+1}\right)\left(X_{i,a}\beta_{m}\right)\\ & \;+\left(\Lambda ⊗I_{J+1}\right)\left(Z_{i}v_{\mathrm{mi}}^{a}+\epsilon_{\mathrm{mi}}^{a}\right)+b_{\mathrm{mi}}^{a}⊗1_{J+1}+e_{\mathrm{mi}}^{a}. \end{array}$$

By combining random effects and error terms in both the level‐1 model and level‐2 model, the marginal distribution for $\left\{\left.Y_{i}^{a}\right|C_{i}=m,X_{i}\right\}$ can be obtained as

(6) $$\left.{Y_{i}}^{a}\right|C_{i}=m,X_{i}∼MVN_{\beta_{m},\lambda_{m0},\lambda ,\sigma_{v},\psi_{m}}\left(\mu_{\mathrm{mi}}^{a},\sum_{mi}\right),$$

where $\mu_{\mathrm{mi}}^{a}=\lambda_{m0}⊗1_{J+1}+(\Lambda ⊗I_{J+1})(X_{i,a}\beta_{m})$, $\sum_{mi}=(\Lambda ⊗I_{J+1})Z_{i}\sum_{v}{\left[(\Lambda ⊗I_{J+1})Z_{i}\right]}^{T}+(\Lambda ⊗I_{J+1}){\left(\Lambda ⊗I_{J+1}\right)}^{T}+diag(\xi_{m1},\ldots ,\xi_{mK})⊗(1_{J+1}1_{J+1}^{T})+diag(\tau_{m1}^{2},\ldots ,\tau_{mK}^{2})⊗I_{J+1}$. $\sum_{v}$ is the variance‐covariance matrix of random effects $v_{\mathrm{mi}}^{a}$ and $\sigma_{v}$ is the collection of unique parameters in $\sum_{v}$. λ is the collection of all the elements in matrix Λ. $\psi_{m}={\left(\xi_{m1},\ldots ,\xi_{mK},\tau_{m1}^{2},\ldots ,\tau_{mK}^{2}\right)}^{T}$. $X_{i}$ is a vector collecting all explanatory variables.

Here, we assume that potential outcomes $Y_{\mathrm{ij}}^{1}$ and $Y_{\mathrm{ij}}^{0}$ are independent given compliance type, covariates and parameters. Given that both $y_{\mathrm{ij}}^{1}$ and $y_{\mathrm{ij}}^{0}$ are never observed at the same time, the likelihood function of the observed data does not depend on the correlation between potential outcomes. Thus, the correlation between potential outcomes is unimportant under the likelihood‐based approach (see Page 181 in Chapter 8 of Imbens and Rubin [20], Hirano et al. [16]).

#### 2.2.3 Model for Compliance Type $C_{i}$

For the probability of being a complier, we use the following logistic regression model

(7) $$p_{ci}=Pr\left(\left.C_{i}=c\;\right|\;W_{i}=w_{i},\eta \right)=\frac{exp\left({w_{i}}^{\prime }\eta \right)}{1+exp\left({w_{i}}^{\prime }\eta \right)},$$

where $W_{i}$ is the collection of baseline covariates for individual i and η is the collection of coefficients for corresponding covariates.

## 3 Inference

Based on $A_{\mathrm{obs}}$ and $D_{\mathrm{obs}}$, there are three possible observed patterns of $\left(a_{i},d_{i}\right)$: (1,1), (1,0), and (0,0). Let S(1,1), S(1,0) and S(0,0) denote the subsets of units exhibiting each pattern separately. This implies that S(1,1) and S(1,0) include the compliers and never‐takers, respectively, in the treatment group and S(0,0) represents a mixture of compliers and never‐takers in the control group. Let $\pi =(\beta_{c},\beta_{n},\lambda ,\lambda_{c0},\lambda_{n0},\sigma_{v},\psi_{c},\psi_{n},\eta )$, the likelihood function based on observed data for all participants in the study is

(8) $$\begin{array}{cc} & ℒ\left(\left.\pi ;Y_{\mathrm{obs}},D_{\mathrm{obs}},A_{\mathrm{obs}}\;\right|\;X\right)\\ & \;=\underset{i}{\prod }\int \;\int \;\int \;\int f\left(\left.y_{i}^{1},y_{i}^{0}\right|U_{\mathrm{mi}}^{1},U_{\mathrm{mi}}^{0},D_{i}(1),D_{i}(0),X_{i};\lambda ,\lambda_{c0},\lambda_{n0},\psi_{c},\psi_{n}\right)\\ & \;f\left(\left.U_{\mathrm{mi}}^{1},U_{\mathrm{mi}}^{0}\right|D_{i}(1),D_{i}(0),X_{i};\beta_{c},\beta_{n},\sigma_{v}\right)f\left(\left.D_{i}(1),D_{i}(0)\right|W_{i};\eta \right)\\ & \;d\;U_{\mathrm{mi}}^{1}d\;U_{\mathrm{mi}}^{0}d\;Y_{i}^{\mathrm{mis}}d\;D_{i}^{mis}\\ & \;=\underset{i}{\prod }\int \;\int f\left(\left.y_{i}^{1},y_{i}^{0}\right|D_{i}(1),D_{i}(0),X_{i};\beta_{c},\beta_{n},\lambda ,\lambda_{c0},\lambda_{n0},\sigma_{v},\psi_{c},\psi_{n}\right)\\ & \;f\left(\left.D_{i}(1),D_{i}(0)\right|W_{i};\eta \right)d\;Y_{i}^{\mathrm{mis}}d\;D_{i}^{mis}\\ & \;=L_{11}\times L_{10}\times L_{00}, \end{array}$$

where

$$\begin{array}{cc} L_{11} & =\underset{\left\{i\in S(1,1)\right\}}{\prod }p_{ci}\frac{1}{{\left(2\pi \right)}^{\frac{J(K+1)}{2}}{\left|\sum_{ci}\right|}^{\frac{1}{2}}}\exp\\ & \;\left\{-\frac{1}{2}{\left(y_{\mathrm{obs},\;i}-\mu_{\mathrm{ci}}^{1}\right)}^{T}\sum_{ci}^{-1}\left(y_{\mathrm{obs},\;i}-\mu_{c_{i}}^{1}\right)\right\},\\ L_{10} & =\underset{\left\{i\in S(1,0)\right\}}{\prod }(1-p_{ci})\frac{1}{{\left(2\pi \right)}^{\frac{J(K+1)}{2}}{\left|\sum_{ni}\right|}^{\frac{1}{2}}}\exp\\ & \;\left\{-\frac{1}{2}{\left(y_{\mathrm{obs},\;i}-\mu_{\mathrm{ni}}^{1}\right)}^{T}\sum_{ni}^{-1}\left(y_{\mathrm{obs},\;i}-\mu_{\mathrm{ni}}^{1}\right)\right\},\\ L_{00} & =\underset{\left\{i\in S(0,0)\right\}}{\prod }\left[p_{ci}\frac{1}{{\left(2\pi \right)}^{\frac{J(K+1)}{2}}{\left|\sum_{ci}\right|}^{\frac{1}{2}}}\exp\right.\\ & \;\left\{-\frac{1}{2}{\left(y_{\mathrm{obs},\;i}-\mu_{c_{i}}^{0}\right)}^{T}\sum_{ci}^{-1}\left(y_{\mathrm{obs},\;i}-\mu_{c_{i}}^{0}\right)\right\}\\ & \;\;\;+(1-p_{ci})\frac{1}{{\left(2\pi \right)}^{\frac{J(K+1)}{2}}{\left|\sum_{ni}\right|}^{\frac{1}{2}}}\exp\\ & \;\left.\left\{-\frac{1}{2}{\left(y_{\mathrm{obs},\;i}-\mu_{\mathrm{ni}}^{0}\right)}^{T}\sum_{ni}^{-1}\left(y_{\mathrm{obs},\;i}-\mu_{\mathrm{ni}}^{0}\right)\right\}\right]. \end{array}$$

$\mu_{\mathrm{mi}}^{a}$ and $\sum_{mi}$ are defined as shown in Equation (6). By combining the level‐1 and level‐2 models, we obtain the above closed‐form simplified marginal likelihood that integrates out the latent factors $U_{\mathrm{mi}}^{a}$. That is, in the likelihood function (Equation (8)), the second equality holds by applying the conclusion shown in Equation (6).

The joint log‐likelihood function for all model parameters using observed data can be maximized using the Quasi‐Newton algorithm, as implemented in the R function optim(). This yields the maximum likelihood estimates (MLEs) of all model parameters simultaneously. Under standard regularity conditions, the MLEs are consistent and asymptotically normal. The asymptotic variance‐covariance matrix of the MLEs is given by the inverse of the Fisher information matrix evaluated at the true parameter values, and in practice can be estimated by the inverse of the observed information matrix, that is, the negative Hessian of the log‐likelihood function evaluated at the MLEs.

When missing data occur for reasons such as attrition or nonresponse, the likelihood specified above for the latent‐factor MCACE model assumes the standard missing at random (MAR) mechanism, under which the probability of missingness depends only on observed data. Consequently, valid likelihood‐based inference does not require explicit modeling of the missingness mechanism. In the AHJ study, the MAR assumption implies that missingness is independent of unobserved outcomes, conditional on observed outcomes and fully observed baseline covariates. Because the proposed MCACE approach is likelihood‐based, it yields valid inference under the MAR assumption.

## 4 Simulation Study

In this section, we conduct simulation studies to examine the performance of the model proposed in Section 2. When generating the simulated dataset, we consider the following level‐1 model with two (Q=2) latent factors ($U_{1mij}^{a}$ and $U_{2mij}^{a}$):

(9) $$\begin{array}{ll} & \left.y_{ijk}^{a}\;\right|\;\left(C_{i}=m,U_{1mij}^{a},U_{2mij}^{a},b_{mik}^{a}\right)\\ & \;=\lambda_{m0k}+\lambda_{k1}U_{1mij}^{a}+\lambda_{k2}U_{2mij}^{a}+b_{mik}^{a}+e_{mijk}^{a}, \end{array}$$

with the following level‐2 model for the $q^{th}$ latent factor $U_{qmij}^{a}$:

(10) $$\begin{array}{ll} U_{qmij}^{a} & =\beta_{qm10}t_{ij}+\beta_{qm20}t_{ij}^{2}+\beta_{qm01}D_{i}(a)\\ & \;+\beta_{qm11}D_{i}(a)t_{ij}+\beta_{qm21}D_{i}(a)t_{ij}^{2}+v_{qm1i}^{a}t_{ij}+\epsilon_{qmij}^{a}, \end{array}$$

where the index of latent factor q=1 or 2. For these two latent factors, we assume that the covariates for fixed effects include the linear time trend ($t_{ij}$), quadratic time trend ($t_{ij}^{2}$), treatment receipt $D_{i}(a)$, and the interactions between the receipt of treatment and these time trends ($D_{i}(a)t_{ij}$ and $D_{i}(a)t_{ij}^{2}$). Specifically, the term $\beta_{qm01}D_{i}(a)$ captures the mean baseline differences in the $q^{th}$ latent factor within compliance type m between the treatment group and the control group. For a randomized controlled trial, the baseline difference within compliance type m between treatment group and control group is expected to be negligible for two factors. Therefore, we set $\beta_{qm01}=0$ for q=1 or 2 in the simulation studies. To simplify the simulation setting and for the comparison convenience, we did not incorporate additional baseline covariates in the level‐2 model. And the covariates for random effects $v_{\mathrm{qmi}}^{a}$ contain linear trend only. For the sake of identification, the intercept and random intercept are removed from the level‐2 model, given that these parameters already appear in the level‐1 model (Assumption 8 in Table 1). When generating the compliance status $C_{i}$ in Equation (7), we considered a binary covariate generated from a Bernoulli distribution. More details regarding the simulation setting can be found in the Supporting Information.

We evaluated the performance of the model proposed under both confirmatory and exploratory analyses. The results regarding model performance are presented in the Supporting Information. These results show that model parameters can be recovered very well under both confirmatory and exploratory analyses. We also examined how well the model recovers the latent structure. For both confirmatory and exploratory analysis, the sample means of the estimates for the entries in the factor loading matrix are close to their corresponding true values, and the sample means of their standard error estimates obtained by using the Fisher information closely approximate their corresponding sample standard deviations of parameter estimates. Furthermore, the empirical coverage probabilities of the 95% confidence intervals, constructed using standard error estimates based on the Fisher information, are approximately at the nominal coverage level. Overall, the method proposed recovers the latent structure well under both confirmatory and exploratory analyses (Supporting Information Tables 1 and 2).

A key focus here is a power analysis to compare the latent‐factor multivariate CACE analysis with the univariate CACE analysis regarding statistical power to detect the intervention effects on multiple study endpoints. Rather than analyzing multiple endpoints jointly, as in latent‐factor multivariate CACE analysis, univariate CACE analysis is conducted for each endpoint separately. Thus, univariate CACE analysis ignores the potential correlation among multiple outcomes collected simultaneously. We describe in detail the model specification for the univariate CACE analysis in the Supporting Information. The data is generated as described above except for comparison convenience, we set $\beta_{qc21}=0$ for q=1,2 (Equation (10)). In the compliance model (Equation (7)), we consider intercept only and set $p_{ci}=p_{c}=0.3$. Both the latent‐factor multivariate CACE analysis and the univariate CACE analysis include $t_{ij}$, $t_{ij}^{2}$ and $D_{i}(a)t_{ij}$ as predictors in the model. For compliers, the coefficient for $D_{i}(a)t_{ij}$ is the difference in outcomes between the treatment group and the control group, which captures the CACE in univariate CACE analysis. In the latent‐factor MCACE analysis, one single global likelihood ratio test is conducted. The null hypothesis for the global test is $\beta_{qc11}=0$ for q=1,2, which means there are no treatment effects for both latent factors among compliers. For univariate CACE analysis, the outcome‐specific null hypothesis is that the coefficient of the predictor $D_{i}(a)t_{ij}$ equals 0 for a specific outcome, which implies that no treatment effect is detected for this outcome. As such, univariate CACE analysis implements six likelihood ratio tests for each outcome because these outcomes are analyzed separately. Under the latent factor model in the simulation study, the null hypothesis for MCACE is equivalent to an overall null hypothesis of no treatment effects for all six outcomes in univariate CACE analysis. Because power is defined as the probability of rejecting the null hypothesis when the null hypothesis is false, we calculate the proportion of rejecting the null hypothesis over 500 simulated datasets. The significance level for the single global test of the null hypothesis of no intervention effects on both latent factors is set as 0.05 for latent‐factor multivariate CACE analysis. For univariate CACE analysis, the significance level for each individual test is adjusted as 0.05/K, where K=6 because there are 6 tests. This adjustment is based on the Bonferroni correction to control the overall familywise Type I error rate at the 0.05 level with multiple testing. When calculating the power for univariate analysis, the null hypothesis is rejected if treatment effect is detected for at least one outcome at a significance level of 0.05/6.

**FIGURE 2:**
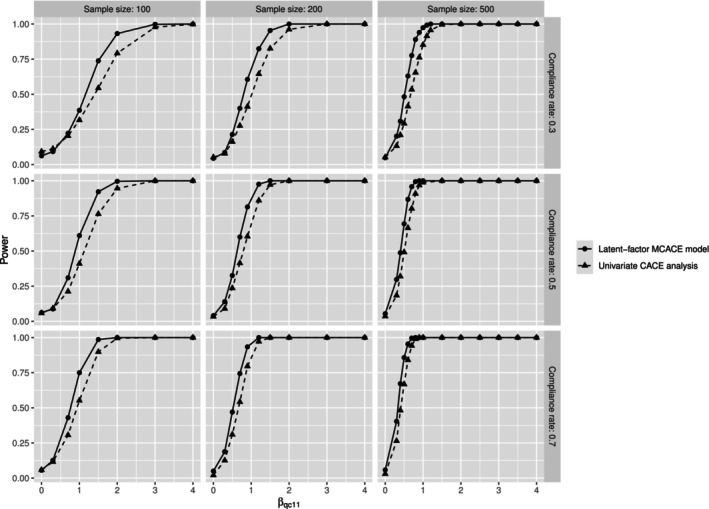
Power analysis, based on 500 simulated datasets.

Figure 2 presents the power curves for the latent‐factor multivariate CACE model and univariate CACE analysis as the sample size varies from 100 to 500 and compliance rate ranges from 0.3 to 0.7. To ease the result presentation, we set the values of $\beta_{qc11}$ across q to be the same and the common value varies from 0 to 4. When $\beta_{qc11}=0$ for any q, the probability of rejecting the null hypotheses is the Type I error rate. We observe the Type I error rates are around 0.05 for the latent‐factor multivariate CACE model across different scenarios (Figure 2), which means Type I error rates remain well controlled under the latent‐factor MCACE model. In contrast, the statistically inefficient univariate CACE analysis exhibits unstable Type I error rates, fluctuating around the nominal 0.05 level depending on sample size and compliance rate. For example, when sample size is relatively small (100) and compliance rate is low (0.3), the Type I error rate is not well controlled and inflates to 0.092. As the value of $\beta_{qc11}$ increases, we observe that the power of the latent‐factor multivariate CACE model (the solid line in Figure 2) approaches 1 much more rapidly than that of the univariate CACE model (the dashed line in Figure 2), especially under small sample size and low compliance rate. For instance, in the scenario where sample size equals 100 and compliance rate is 0.3, the power increases from 0.544 under the univariate CACE analysis to 0.738 under the latent‐factor multivariate CACE model when $\beta_{qc11}=1.5$. These results demonstrate that the latent‐factor MCACE model outperforms univariate CACE analysis in terms of both statistical power and Type I error control for hypothesis testing. This advantage stems from the fact that univariate CACE analysis fails to account for correlations among multiple endpoints, whereas the latent‐factor multivariate CACE approach incorporates these correlations through latent factors, thereby leveraging this additional information to yield more efficient and stable inference and to enhance study power.

## 5 Application

### 5.1 Study Description

We applied the proposed model to the Arthritis Health Journal (AHJ) study. AHJ is a patient‐centered online tool that supports rheumatoid arthritis (RA) patients in managing their disease by actively monitoring their symptoms and tracking disease activity. A total of 94 participants were recruited and randomly assigned to the treatment group (*n* = 45) and the control group (*n* = 49). Patients in the treatment group received access to the AHJ tool immediately, while patients in the control group had to wait for six months before being granted access to the tool. The study's primary analysis focused on the data from the first six months. Some participants randomized to the treatment group may not have used the tool or perhaps used it only rarely which constituted noncompliance behavior. Discussions among doctors and patients indicate that it is necessary to use the tool at least once per month for it to produce an effect. Therefore, compliers are defined as patients who would use the tool at least one time per month on average if assigned to the treatment group [7].

Participants were evaluated by online questionnaires every three months (i.e., at baseline, the third month, and the sixth month). Baseline questionnaires collected information about demographics and disease information. Follow‐up questionnaires evaluated the frequency with which the participants used the tool, consumer effectiveness attributes, self‐efficacy and satisfaction with care. In this study, six endpoints were collected to evaluate the tool's treatment efficacy with respect to the underlying constructs (i.e., self‐efficacy and satisfaction with care): **(1) effective consumer 17 scale**, the average score of 17 items concerning how participants manage their disease on a scale from 0 to 100, where 100 indicates “most confident”; **(2) manage symptoms scale**, the average score of 5 items concerning how patients manage their symptoms on a scale from 0 to 10, where 10 indicates “totally confident”; **(3) manage disease in general scale**, the average score of 5 items concerning how patients manage their disease in general on a scale from 0 to 10, where 10 indicates “totally confident”; **(4) communicate with physician scale**, the average score of three items concerning patients' confidence in communicating with their rheumatologists on a scale from 0 to 10, where 10 indicates “totally confident”; **(5) partners in health scale**, the average score of 11 items concerning patients' knowledge of disease and treatment on a scale from 0 to 80, where 80 indicates “poor self‐management”; **(6) satisfaction with various aspects of medical care**, the average score of 8 items concerning their satisfaction with various aspects of medical care on a scale from 0 to 10 scale, where 10 indicates “completely satisfied”. All six endpoints are the averages of several individual items and consequently take continuous values. Six outcomes are rescaled to a 0 to 100 scale so that these endpoints have comparable variances. The direction of the fifth outcome is also adjusted so that a higher value represents a beneficial result for all these six endpoints. The rescaling of outcomes eases the interpretation of estimation results but does not influence statistical inference. Furthermore, although the number of participants is only moderately large (*n* = 94), six outcomes were collected at three time points for participants in this study, making a total of eighteen individual‐level outcomes to be measured. Under the MCACE model, all these observations (six outcomes across three time points per person) are considered jointly. Therefore, the total number of observations used to estimate the model is considerably greater than the number of study participants.

All baseline covariates and baseline outcome measurements were fully observed. Missingness for outcome measurements occurred during the follow‐up period. At the third month, 11 participants (24.4%) in the treatment arm and 3 participants (6.1%) in the control arm had missingness in outcome measurements. At the sixth month, 10 participants (22.2%) in the treatment arm and 7 participants (14.3%) in the control arm had missingness in the outcomes. The MCACE analysis employed a likelihood‐based approach, which allows for valid inference under the missing at random (MAR) mechanism, a less restrictive assumption than missing completely at random (MCAR).

### 5.2 Latent‐Factor MCACE Analysis

This analysis aims to (1) evaluate the AHJ tool's effectiveness on underlying constructs (e.g., patients' self‐efficacy in disease management and the effectiveness in shared decision‐making); and (2) determine how covariates predict the compliance behavior. We summarize the implementation of the proposed method in three main steps: (i) determining the appropriate number of factors, (ii) specifying the factor loading structure for the multivariate outcomes, and (iii) conducting model selection.

First, it is necessary to restrict the range of possible models (e.g., the number of latent factors). Choosing the number of factors is among the most critical and debated steps in factor analysis. There is no single definitive rule; instead, researchers typically rely on a combination of statistical criteria, substantive theory, and interpretability. Retained factors should make conceptual sense and ideally exhibit a simple structure (i.e., outcomes load strongly on one factor and weakly on others). In some cases, prior theory or earlier studies provide clear guidance on the number of latent constructs. In the AHJ study, the design of the multivariate outcomes and preliminary inspection of the data suggested a reasonable range for the number of latent factors, as described later. In other settings where prior knowledge is limited, standard approaches from traditional factor analysis may be considered to inform the plausible range of models. For example, a scree diagram of eigenvalues computed from outcomes at baseline can be used, with the maximum number of factors chosen at the “elbow” of the plot [21].

Second, given a specified number of factors, it is important to determine the structure of the factor loading matrix. In the absence of prior knowledge about the scientific relationships between outcomes and latent constructs, exploratory methods are required, often imposing constraints such as $\lambda_{kq}=0$ when q>k to ensure identifiability. In the AHJ study, however, we had substantive prior knowledge regarding the interpretation of the six outcomes (e.g., observed trends, scientific rationale). This allowed us to specify the loading structure directly and conduct a confirmatory analysis.

Finally, model selection is guided by both statistical and substantive considerations. Over‐extraction of factors can lead to uninterpretable and fragmented constructs, while under‐extraction risks omitting meaningful latent dimensions. Commonly used statistical tools include likelihood ratio tests, the Akaike Information Criterion (AIC) [22], and the Bayesian Information Criterion (BIC) [23], with models yielding lower AIC and BIC values generally preferred. It is advisable to examine and compare the CACE estimates across alternative model specifications. Overall, comparing competing models while balancing statistical fit, parsimony, interpretability, and theoretical coherence is essential.

**FIGURE 3:**
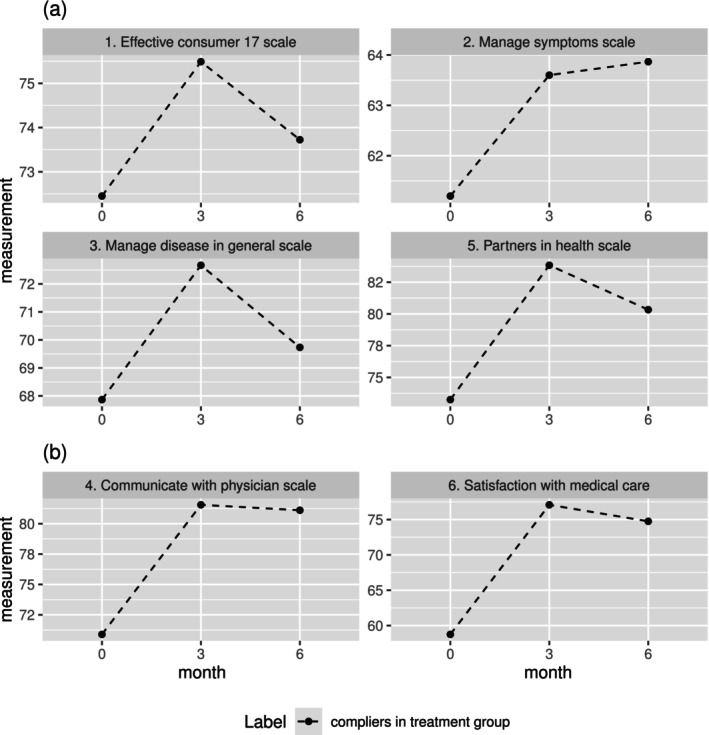
(a) shows outcome means in the compliers in the treatment group for the 1st, 2nd, 3rd, and 5th endpoints; (b) shows outcome means for the 4th and 6th endpoints.

In the AHJ study, the characteristics of six endpoints suggest some possible latent structures. First, because the six endpoints capture different perspectives of the effect of using the tool, it may be a good starting point to consider only one latent factor. Under this assumption, all six endpoints load on a common latent factor, which captures the overall treatment effect. Thus, we can determine whether the AHJ helps patients manage their disease or not based on the common latent factor. Alternatively, the design of these six endpoints suggests the other more plausible structure which involves two latent factors. Substantively, in the AHJ study, effective consumer 17 scale (the first endpoint), manage symptoms scale (the second endpoint), manage disease in general scale (the third endpoint), and partners in health scale (the fifth endpoint) were used to measure self‐efficacy. Interaction with health care providers was measured by the communicate with physician scale (the fourth endpoint) and satisfaction with various aspects of medical care (the sixth endpoint). These endpoints' two‐group structure based on their substantive meanings is largely consistent with the trends illustrated in Figure 3, which justifies a model with two latent constructs. Figure 3a shows the outcome means in the compliers in the treatment group for the first three endpoints and the fifth endpoint. Figure 3b shows the outcome means for the fourth and sixth endpoints. We observe that the outcome trajectories over time for the compliers in the treatment group are similar among endpoints within each of the two panels (a and b) of Figure 3. It is worth noting that the trends for the fourth and sixth endpoints are almost identical in Figure 3b. In Figure 3a, the trends are similar across all outcomes except the second. However, the second outcome is still grouped into the Figure 3a because it is closely related to self‐efficacy conceptually. Specifically, in a confirmatory factor analysis, the fourth and sixth outcomes can be grouped together to indicate one latent construct that measures the participants' knowledge and the ability to interact with health care providers. The remaining endpoints are grouped to indicate the other latent construct that measures self‐efficacy in disease management. Therefore, the first three endpoints and the fifth endpoint load on the first latent factor, while the fourth and sixth endpoints load on the second latent factor. Thus, when considering two latent factors, the level‐1 model is specified as Equation (1) where Q=2 and K=6, with the following factor loading matrix:

(11) $$\Lambda^{\prime }=\left(\begin{array}{cccccc} \lambda_{11} & \lambda_{21} & \lambda_{31} & 0 & \lambda_{51} & 0\\ 0 & 0 & 0 & \lambda_{42} & 0 & \lambda_{62} \end{array}\right).$$

The level‐2 model is specified as Equation (2) where quadratic time trends are included (shown in Equation (12) below):

(12) $$\begin{array}{ll} U_{qmij}^{a} & =\beta_{qm10}t_{ij}+\beta_{qm20}t_{ij}^{2}+\beta_{qm01}D_{i}(a)+\beta_{qm11}D_{i}(a)t_{ij}\\ & \;+\beta_{qm21}D_{i}(a)t_{ij}^{2}+v_{qm1i}^{a}t_{ij}+v_{qm2i}^{a}t_{ij}^{2}+\epsilon_{qmij}^{a}. \end{array}$$

The quadratic time trend model provides a flexible specification and serves as a saturated model for the three visit time points in the AHJ study, thereby minimizing the risk of misspecifying the trajectories of the latent factors. Within the level‐2 model, we set the baseline difference between compliers in the treatment group and compliers in the control group at null ($\beta_{qc01}=0$ for q=1 or 2 in Equation (12)), given that this is a randomized controlled trial. Furthermore, the random effects for the second latent factor are removed ($v_{2m1i}^{a}=0$ and $v_{2m2i}^{a}=0$ for m=c or n in Equation (12)) based on the likelihood ratio test [24].

The compliance model is specified as stated in Equation (7). The compliance model (Equation (7)) includes four binary baseline covariates: early disease (1 indicates early disease (0‐2 years) and 0 indicates late disease (≥ 2 years)); high disease activity (1 indicates high disease activity (high RAPID4 values) and 0 indicates low disease activity (remission, moderate/low RAPID4 values)); male (1 indicates male and 0 indicates female); and older age (1 indicates above the median age (54.5) and 0 otherwise).

Additionally, we also considered a model with three factors. Since the outcome trend for the second outcome (manage symptoms scale) is somewhat different from mean trends for other outcomes in Figure 3a, the first, third, and fifth outcomes are grouped together to represent the underlying construct, self‐efficacy in disease management; the second outcome is grouped separately to indicate a new underlying construct representing itself. Similar to the two‐group structure, the remaining outcomes (fourth and sixth outcomes) are grouped together to indicate the underlying construct, interaction with health care providers. Therefore, the first, third, and fifth outcomes load on the first latent factor, the second outcome loads on the second latent factor, and the remaining outcomes load on the third factor. Table 2 lists the maximums of the log‐likelihood functions, along with the AIC and BIC, for models with varying numbers of factors. It emerges that the model with two latent factors gives the lowest AIC and BIC. Therefore, the two‐factor model is the preferred model, taking into account model fit, parsimony, and interpretability altogether.

**TABLE 2:** Model selection.

Number of factors	logL	AIC	BIC
1	−5915.344	11942.69	12085.11
2	−5897.497	**11918.99**	**12076.68**
3	−5892.786	11921.57	12094.52

Table 3 presents the estimation results obtained under the two‐factor model. As seen from Equation (12), the underlying latent factors are permitted to evolve over time and exhibit substantial heterogeneity in individual‐specific trajectory parameters, such as slope or quadratic trends. However, the interpretation of these factors is governed by the factor loading matrix, which remains invariant across time, compliance types, and individuals. The outcome measures are positively correlated with two latent factors based on the positive estimates of matrix Λ, such that higher factor values indicate better outcomes. Given the involvement of two latent factors, the CACE on each latent factor at the sixth month, the primary time point of the interest in this study, is $𝔼(U_{qmi2}^{1}|m=c)-𝔼(U_{qmi2}^{0}|m=c)=\beta_{qc11}*2+\beta_{qc21}*4$, which is obtained based on Equations (4) and (12) for j=2 and $t_{ij}=2$. According to Table 3, the CACEs on the first and second latent factors are 1.392 (SE = 0.632) and 1.745 (SE = 0.660), respectively. Corresponding *p*‐values are 0.028 and 0.008, respectively, based on the Wald test. Given that the estimates of CACEs for these two latent factors are positive and the *p*‐values are significant, these results suggest that the AHJ had beneficial causal effects on both self‐efficacy and interaction with health care providers among RA patients who comply with the assigned treatments.

**TABLE 3:** Estimates and standard errors for causal treatment effects in the AHJ study.

Two latent factors				
Fixed effects estimation^a^				
Parameter	Estimate	SE	95% Confidence interval	*p*‐value
$\beta_{1c11}$	2.772	1.204	(0.413, 5.132)	0.021
$\beta_{1c21}$	−1.038	0.529	(−2.075, −0.001)	0.050
$\beta_{2c11}$	1.900	1.176	(−0.406, 4.205)	0.106
$\beta_{2c21}$	−0.514	0.582	(−1.653, 0.626)	0.377

Treatment effect estimation at the sixth month				
	Estimate	SE	95% Confidenceinterval	*p*‐value
${\text{CACE}}_{1}$ ^b^	1.392	0.632	(0.153, 2.631)	0.028
${\text{CACE}}_{2}$ ^c^	1.745	0.660	(0.452, 3.039)	0.008

Estimation of factor loading matrix Λ		
Parameter	Estimate	SE
$\lambda_{11}$	7.918	0.683
$\lambda_{21}$	2.698	0.557
$\lambda_{31}$	9.067	0.674
$\lambda_{41}$	—	—
$\lambda_{51}$	8.553	0.722
$\lambda_{61}$	—	—
$\lambda_{12}$	—	—
$\lambda_{22}$	—	—
$\lambda_{32}$	—	—
$\lambda_{42}$	6.524	1.202
$\lambda_{52}$	—	—
$\lambda_{62}$	6.638	1.236

^a^ Fixed effects estimation section only shows parameters related to treatment effect estimation.

^b^ CACE for the first factor, self‐efficacy.

^c^ CACE for the second factor, interaction with health care providers.

We further examine the sensitivity of the CACE estimates to the specification of factor structures by comparing results across models with different numbers of factors. Estimation results for the one‐factor and three‐factor models are reported in Supporting Information Tables 3 and 5, respectively. In all cases, the estimated parameters in the factor loading matrices are positive, indicating that the potential outcomes are positively associated with the latent factors, consistent with the two‐factor specification. For the one‐factor model, the estimated CACE on the common latent factor is 1.447 (SE = 0.636, *p* = 0.023; Supporting Information Table 3), suggesting an overall significant beneficial effect of the AHJ intervention. For the three‐factor model, the results in Supporting Information Table 5 show that all CACE estimates remain positive. The estimated CACE on the first factor (self‐efficacy in disease management), which loads on the first, third, and fifth outcomes, is 1.421 (SE = 0.620, *p* = 0.022). The estimated CACE on the second factor, primarily defined by the second outcome, is 0.437 (SE = 0.865, *p* = 0.613). The estimated CACE on the third factor (interaction with healthcare providers), which loads on the fourth and sixth outcomes, is 1.761 (SE = 0.657, p=0.007). Collectively, these results indicate that the main conclusions are robust to the choice of factor structures, with qualitatively similar findings across specifications.

We are also interested in identifying RA patients who are more likely to be a complier. Table 4 presents the estimation results of the compliance model under the scenario where two latent factors are considered. The coefficients for all covariates except high disease activity are negative. In addition, the coefficients for early disease and high disease activity are statistically significant. The *p*‐value of the coefficient for older age approaches statistical significance. Overall, the estimation results suggest that younger female patients with longer disease duration and high disease activity were more likely to be compliers.

**TABLE 4:** Estimation results of compliance model.

Two latent factors			
Covariates	Estimate	Standard error	*p*‐value
Intercept	−0.825	0.614	0.179
Early disease	−2.389	1.121	0.033
High disease activity	1.420	0.643	0.027
Male	−0.888	0.877	0.311
Older age	−0.938	0.488	0.054

*Note:* Early disease = 1 if early disease (0–2 years) and = 0 if late disease (≥ 2 years), high disease activity = 1 if high disease activity (high RAPID4 values) and = 0 if low disease activity (remission, moderate/low RAPID4 values)), Male = 1 if male and = 0 if female, Older age = 1 if above the median age (54.5) and = 0 if otherwise.

### 5.3 Alternative Analysis

Additionally, we conducted two alternative analyses. First, we demonstrated the visible benefits of using the proposed latent‐factor MCACE approach by conducting univariate CACE analyses for comparison. Second, we assessed the plausibility of the exclusion restriction assumption.

#### 5.3.1 Univariate CACE Analysis

To demonstrate the advantages of the proposed latent‐factor MCACE model, we conducted univariate CACE analyses based on pre‐specified functions of the individual outcome measures (i.e., subscales). Consistent with the two‐factor structure identified in the application section, we constructed two domain scores: the average of the first three and the fifth outcomes, and the average of the fourth and sixth outcomes. Univariate CACE analyses were then conducted for each domain score separately. At six months, the estimated CACE for the first domain score was 5.717 (SE = 3.088), whereas the estimate for the second domain score was 16.150 (SE = 5.138). The corresponding Wald test *p*‐values were 0.064 and 0.002, respectively. The effect estimates from the univariate CACE analyses differ from those obtained from the latent‐factor MCACE model because the MCACE estimates are expressed on the scale of latent factor scores, as defined by the factor loading matrix. Notably, the *p*‐value for the first domain score (self‐efficacy) in univariate CACE analysis is not statistically significant. This lack of significance reflects potential information loss from using simple averaged domain scores, which may not optimally capture the underlying latent structure.

In practice, real‐world RCTs, including the AHJ study, often employ multiple study outcomes to measure treatment effects on a small number of underlying constructs. In such settings, both the univariate CACE analyses based on simple averages of subscales and the latent‐factor MCACE model are motivated by several considerations: (1) improving statistical power and efficiency relative to analyzing each subscale separately; (2) achieving parsimony by reducing high‐dimensional subscales to a lower‐dimensional representation; (3) alleviating multiple testing concerns; and (4) facilitating direct inference on the underlying constructs of scientific interest. For example, in the AHJ study, evaluating the intervention's effects on the latent constructs of self‐efficacy and interaction with health care providers is of primary scientific importance. As shown above, the proposed latent‐factor MCACE approach outperforms the univariate CACE analysis based on prespecified simple averages in two main aspects. First, it makes more efficient use of available information, potentially increasing power to detect treatment effects. In contrast, univariate CACE relies on simple averages of subscales, which impose equal and potentially suboptimal weights, resulting in information loss. Second, the latent‐factor MCACE model explicitly captures the relationships between latent constructs and observed subscales through the factor‐loading matrix. This framework provides a data‐driven approach to deriving subscale weights, rather than relying on ad hoc equal weighting. Consequently, the latent‐factor MCACE model can yield more informative and efficient inference by fully leveraging the available information contained in all observed subscales.

#### 5.3.2 Sensitivity Analysis

One issue that may arise in the MCACE analysis concerns the definition of compliers. This definition is not always clear‐cut in RCTs, and the ambiguity in the definition can challenge the plausibility of the exclusion restriction assumption. The primary goal of the AHJ tool is to enhance patients' self‐management by helping them better understand their disease and seek timely medical assistance. Consistent and regular use of the tool is therefore essential to achieving this objective. As noted earlier, discussions with doctors and patients determined that using the AHJ tool at least six times over six months is required for the tool to produce an effect. Accordingly, we define compliers as patients who would use the tool, on average, at least once per month if assigned to the intervention group. Under this definition, however, never‐takers include participants in the treatment group who use the tool only rarely. Since exclusion restriction implies no difference in potential outcomes under treatment and control assignments for never‐takers, the plausibility of the exclusion restriction assumption may be questioned due to the observed differences in tool usage between never‐takers in the treatment group and never‐takers in the control group.

To address this concern, we performed a sensitivity analysis using two alternative definitions of compliers. Under the first alternative definition (D1), compliers are patients who would use the tool at least three times over six months if assigned to the intervention arm. Under the second definition (D2), compliers are patients who would use the tool at least once over six months. The original definition, D1, and D2 reflect increasing plausibility of the exclusion restriction assumption. In particular, under D2, never‐takers in both the treatment and control groups would have no tool usage, making the exclusion restriction most plausible, though at the cost of a higher risk of misclassifying some patients who used AHJ rarely as compliers. The CACE estimation results under these two alternative definitions are reported in Supporting Information Table 7. For consistency with Section 5.2, we considered the latent‐factor MCACE model with two latent factors: self‐efficacy and interaction with health care providers. The estimates for CACEs are comparable with those obtained under the original definition that compliers are patients who would use the tool at least six times over six months. Across all definitions, the CACE estimates for self‐efficacy remained close to 1.4, ranging from 1.240 to 1.392. The CACE estimates for interaction with health care providers remained around 1.7, ranging from 1.671 to 1.793. These findings indicate that the conclusions of positive and significant CACEs on the two latent factors are robust to different definitions of compliers with varying plausibility for the exclusion restriction assumption.

## 6 Discussion

The proposed model introduced latent factors that capture the underlying constructs driving multiple endpoints measured over time, and yield parsimonious estimation of CACEs on these latent constructs in the presence of treatment noncompliance. The results from simulation studies demonstrate that the proposed model can be identified, and all true values of model parameters are recovered well. Moreover, compared with univariate CACE analysis, the power analysis shows a substantial gain in the study power under the latent‐factor multivariate CACE model. In the application section, we first conducted a model comparison that selected two factors. We then employed the confirmatory analysis and divided the endpoints into two groups based on their characteristics. The first latent factor represents self‐efficacy, while the second measures interaction with healthcare providers. For both latent factors, we detected beneficial CACEs. The estimates from the compliance model show that younger female patients with longer disease duration and high disease activity were more likely to be compliers.

Compared with the prior approach of Guo et al. [7], one advantage of the model proposed here is that we are able to determine the direction of treatment effect (beneficial or not) on the latent factors. Specifically, while the global test of the null hypothesis of no CACEs for all six endpoints conducted in Guo et al. [7] is limited to individual endpoints only, the latent factor approach proposed here yields insights on underlying patients' traits of primary scientific interest and demonstrates that the CACEs on both latent factors are beneficial in the application. Thus, our analysis can directly address the main scientific questions posed by this RCT and yields novel findings not discovered previously. Furthermore, the dimension reduction of our latent‐factor MCACE model can significantly mitigate the multiple testing issues and the loss of statistical power associated with multidimensional outcomes: while hypothesis testing conducted separately for each of the six outcomes by Guo et al. [7] (after rejection of the global test of no CACEs for all six endpoints) could only detect significant CACEs on two of the six outcomes, our latent‐factor MCACE model can detect significant effects on all underlying factors thanks to its more parsimonious and efficient tests.

This study has some limitations. First, the proposed approach is based on latent factor modeling and therefore relies on a combination of statistical criteria, empirical identification of latent factors, and substantive interpretability. As a result, it involves some degree of subjectivity in modeling and model selection. In particular, there is a trade‐off between interpretability and the richness of the latent structure. Higher‐dimensional latent factors may capture more information, but can lead to a fragmented and less interpretable structure. Conversely, a lower‐dimensional specification is more interpretable but may fail to adequately represent meaningful latent constructs. Accordingly, the approach should be implemented carefully and accompanied by rigorous sensitivity analysis. Second, in the methodology proposed, we discuss only the continuous responses and assume the outcomes conditioned on compliance type and covariates follow the multivariate normal distribution. In the future, other distribution assumptions may be considered. In randomized controlled trials, the outcome measures can be highly skewed. In such cases, data transformations (e.g., logarithmic transformation) may be required to better satisfy the normality assumption. Furthermore, exploring sensitivity analyses for outcomes that deviate from normality is important. Potential approaches include extending our level‐1 model using generalized linear mixed models or robust distribution‐free models that accommodate non‐Gaussian outcomes, which can provide complementary robustness checks. Additionally, in real‐life RCTs, we may encounter categorical or mixed‐type responses. While this lies beyond the scope of the present paper, future investigations can consider extending the proposed method to discrete and mixed‐type responses.

## Funding

This work was supported by the BC Academic Health Sciences Network (Grant No. RWCT‐201), NSERC (Grant Nos. RGPIN‐2018‐04313 and RGPIN‐2024‐06629), NIH (Grant No. R01CA178061), and Social Sciences and Humanities Research Council of Canada (Grant Nos. 435‐2018‐0519 and 435‐2023‐0306). The RCT of the Arthritis Health Journal was funded by a Canadian Initiative for Outcomes in Rheumatoid Arthritis (CIORA) grant from the Canadian Rheumatology Association.

## Disclosure

The authors have nothing to report.

## Conflicts of Interest

The authors declare no conflicts of interest.

## Supporting information




**Data S1** Supporting Information.

## Data Availability

The data and code that support the findings in the simulation study are available on request from the corresponding author. The data in the application section are not publicly available due to privacy or ethical restrictions.
