# Supplementary material for: A Latent‐factor MCACE Model for Multidimensional Outcomes and Treatment Noncompliance with Application to a Longitudinal Trial of Arthritis Health Journal
Source: Stat Med. 2026 Apr 26;45:e70532. doi: 10.1002/sim.70532 (PMC13111790; doi:10.1002/sim.70532)
Supplement: Supplementary file 1 — Data S1 Supporting Information. [file SIM-45-0-s001.pdf]

Supporting Information for "A Latent-factor MCACE Model for Multidimensional Outcomes and Treatment Noncompliance with Application to a Longitudinal Trial of Arthritis Health Journal"

Web Appendix A

A.1 Simulation Setting

True values in simulation studies

True values for parameters in Eqns 9 and 10 are listed as below:

$\boldsymbol{\lambda_{c0}} = (58, 60, 61, 59, 60, 58)^T$,

$\boldsymbol{\lambda_{n0}} = (79, 88, 79, 87, 82, 88)^T$,

$$\Lambda = \begin{pmatrix} 7 & 0 \\ 3 & 0 \\ 8 & 0 \\ 0 & 6 \\ 8 & 0 \\ 0 & 5 \end{pmatrix},$$

$\boldsymbol{\beta_c} = (\beta_{1c10}, \beta_{1c20}, \beta_{1c11}, \beta_{1c21}, \beta_{2c10}, \beta_{2c20}, \beta_{2c11}, \beta_{2c21})^T = (-3, 1, 0.5, -1, 2, -1, -1, 1)^T$,

$\boldsymbol{\beta_n} = (\beta_{1n10}, \beta_{1n20}, \beta_{1n11}, \beta_{1n21}, \beta_{2n10}, \beta_{2n20}, \beta_{2n11}, \beta_{2n21})^T = (-3, 2, 0, 0, -2, 1, 0, 0)^T$,

$var(v^a_{1m1i}) = exp(1)$, $var(v^a_{2m1i}) = exp(1.5)$,

$\boldsymbol{\psi_c} = (\xi_{c1}, \cdots, \xi_{c6}, \tau^2_{c1}, \cdots, \tau^2_{c6})^T = (exp(3), exp(5), exp(3), exp(4), exp(3), exp(4), exp(4), exp(4), exp(4), exp(5), exp(4), exp(5))^T$,

$\boldsymbol{\psi_n} = (\xi_{n1}, \cdots, \xi_{n6}, \tau^2_{n1}, \cdots, \tau^2_{n6})^T = (exp(3), exp(4), exp(3), exp(4), exp(3.5), exp(3), exp(5), exp(4), exp(5), exp(4), exp(4), exp(5))^T$.

We include a binary covariate generated from a Bernoulli distribution with probability 0.7. In the compliance model (Eqn 7), the intercept is set to $\eta_0 = -2$, and the coefficient for the covariate is $\eta_1 = 1.5$. These parameter values are chosen so that the marginal compliance rate is approximately 0.3, aligning with the AHJ application.

Data generating process and partial results in simulation studies

In the simulation study, six outcomes were simulated at three time points for each individual based on the model in Eqns 9 and 10. For the factor loading matrix Λ, we set the first, second, third and fifth outcomes to load on the first factor and the remaining two outcomes to load on the second factor in the process of generating the data. This means the fourth and the sixth elements of the first column and the first three elements and the fifth element of the second column of the factor loading matrix (Λ) are fixed at 0, as shown below:

$$\Lambda = \begin{pmatrix} \lambda_{11} & 0 \\ \lambda_{21} & 0 \\ \lambda_{31} & 0 \\ 0 & \lambda_{42} \\ \lambda_{51} & 0 \\ 0 & \lambda_{62} \end{pmatrix}. \tag{S1}$$

Under the setting, we conducted two simulation studies to examine the performance of the proposed model. In the first simulation study, we focused on confirmatory analysis, meaning that the structure of the factor loading matrix Λ was known before the data analysis was conducted. This occurs when researchers have the knowledge of which outcomes load on a specific factor, although the factor loading values are unknown and must be estimated.

In the second simulation study, we focus on exploratory analysis. Under exploratory

analysis, we assume no prior knowledge regarding which outcomes load on each factor. To fix the rotation of the matrix Λ, we imposed the restriction of $\lambda_{kq} = 0$, $q > k$ as noted. That is, the exploratory factor analysis fixes λ_{12} as 0 as shown in Eqn S2.

$$\Lambda = \begin{pmatrix} \lambda_{11} & 0 \\ \lambda_{21} & \lambda_{22} \\ \lambda_{31} & \lambda_{32} \\ \lambda_{41} & \lambda_{42} \\ \lambda_{51} & \lambda_{52} \\ \lambda_{61} & \lambda_{62} \end{pmatrix}. \tag{S2}$$

When estimating the parameters in the factor loading matrix Λ in Eqns $S1$ and $S2$, one element in each column should be restricted to being positive so that parameters can be completely identifiable. Here we assume that λ_{11} and λ_{62} are positive. Otherwise, considering $-\Lambda$ and $-\boldsymbol{U}^{\boldsymbol{a}}_{\boldsymbol{mij}}$ gives the same value of the likelihood function.

Based on Eqn 10, the principal causal effects (PCEs) on latent factors $U^a_{qmij} (q = 1, 2)$ within compliance pattern $C_i = c$ are

$$E(U^1_{qcij}|C_i = c) - E(U^0_{qcij}|C_i = c) = \beta_{qc01} + \beta_{qc11} t_{ij} + \beta_{qc21} t^2_{ij}.$$

Because $\beta_{qc01} = 0$ for $q = 1, 2$ in the simulation setting, β_{qc11} and β_{qc21} jointly capture the PCEs. Thus, we evaluate the performance of the proposed procedure for estimating β_{qc11} and β_{qc21}. In addition, we evaluate the estimation accuracy of the elements in the factor loading matrix (i.e., λ_{k1}, λ_{k2}). Web Table 1 and Web Table 2 report the results relating to parameters β_{qc11} and β_{qc21} for the confirmatory and exploratory analyses, respectively. In both tables, results are obtained based on 500 repetitions when sample size equals 500. We observe sample means of parameter estimates are close to their corresponding true values. Sample means of standard error estimates obtained by using the Fisher information are

close to their corresponding sample standard deviations of estimates. This means model parameters can be recovered very well under both confirmatory analysis and exploratory analysis.

Web Table 1: Estimation accuracy under confirmatory analysis based on 500 repetitions when sample size equals 500

Parameter	True value	Sample mean	$sse^{\dagger}$ ($sm_{se}^{\ddagger}$)	Empirical coverage probability
		Treatment effect		
β_{1c11}	0.5	0.497	0.477 (0.481)	95.4%
β_{1c21}	-1	-1.009	0.214 (0.215)	94.4%
β_{2c11}	-1	-1.024	0.748 (0.768)	95.2%
β_{2c21}	1	1.020	0.361 (0.365)	95.6%
		Factor loading matrix Λ		
λ_{11}	7	6.992	0.264 (0.259)	94.8%
λ_{21}	3	2.996	0.137 (0.133)	95.0%
λ_{31}	8	7.986	0.293 (0.290)	95.0%
λ_{41}	0	-	-	-
λ_{51}	8	7.991	0.284 (0.286)	95.4%
λ_{61}	0	-	-	-
λ_{12}	0	-	-	-
λ_{22}	0	-	-	-
λ_{32}	0	-	-	-
λ_{42}	6	5.933	0.529 (0.506)	95.2%
λ_{52}	0	-	-	-

λ_{62}	5	4.930	0.429 (0.413)	95.2%
		Coefficients in compliance model		
η_0	-2	-2.027	0.256 (0.260)	97.2%
η_1	1.5	1.520	0.283 (0.283)	96.0%

† sse: sample standard deviation of estimates

‡ sm_{se}: sample mean of standard error estimates obtained based on the Fisher information

Web Table 2: Estimation accuracy under exploratory analysis based on 500 repetitions when sample size equals 500

Parameter	True value	Sample mean	$sse^{\dagger}$ ($sm_{se}^{\ddagger}$)	Empirical coverage probability
		Treatment effect		
β_{1c11}	0.5	0.506	0.494 (0.480)	94.0%
β_{1c21}	-1	-1.013	0.225 (0.215)	91.8%
β_{2c11}	-1	-1.015	0.783 (0.764)	94.0%
β_{2c21}	1	1.008	0.364 (0.366)	95.4%
		Factor loading matrix Λ		
λ_{11}	7	6.978	0.274 (0.258)	95.4%
λ_{21}	3	2.996	0.140 (0.133)	94.6%
λ_{31}	8	7.974	0.306 (0.290)	92.0%
λ_{41}	0	-0.023	0.305 (0.294)	94.0%
λ_{51}	8	7.985	0.303 (0.285)	93.8%
λ_{61}	0	-0.033	0.266 (0.264)	95.4%
λ_{12}	0	-	-	-

λ_{22}	0	0.008	0.111 (0.104)	95.0%
λ_{32}	0	0.004	0.162 (0.161)	95.0%
λ_{42}	6	5.956	0.535 (0.506)	93.2%
λ_{52}	0	0.014	0.151 (0.149)	94.6%
λ_{62}	5	4.940	0.440 (0.412)	93.4%
		Coefficients in compliance model		
η_0	-2	-2.033	0.280 (0.261)	95.4%
η_1	1.5	1.536	0.290 (0.284)	95.8%

† *sse*: sample standard deviation of estimates

‡ sm_{se}: sample mean of standard error estimates obtained based on the Fisher information

Web Table 1 and Web Table 2 also show the estimation results for the two-factor loading matrix. For both confirmatory and exploratory analysis, the sample means of the estimates for the entries in the loading matrix are close to their corresponding true values and sample means of their standard error estimates obtained by using the Fisher information approximate their corresponding sample standard deviations of estimates. The last column, "Empirical coverage probability", shows that 95% confidence intervals constructed using standard error estimates based on the Fisher information achieve the nominal 95% coverage rate. In exploratory analysis, since we impose less restriction on Λ and only restrict $\lambda_{kq} = 0$ when $q > k$ to fix the rotation of the factor loading matrix Λ, the matrix Λ produced based on this restriction may be hard to interpret. In this case, factor rotation techniques are often used to improve the interpretability of the factor loading matrix. There are two types of rotation: orthogonal rotation and oblique rotation (Everitt and Hothorn, 2011). Orthogonal rotation creates uncorrelated rotated factors, while oblique

rotation allows correlated factors. For orthogonal rotation, varimax rotation is commonly used. For oblique rotation, oblimin and promax rotation are commonly used. Typically, the aim is to find a rotation matrix which ensures each endpoint has a high loading on just one latent factor. These rotation techniques can be applied to achieve a simple structure which allows the rotated matrix to be more interpretable. In our simulation study, we have applied varimax rotation of the estimated loading matrix in the exploratory analysis.

It is worth noting the benefits of confirmatory analysis where the structure of factor loading matrix is known. Compared to exploratory analysis (Web Table 2), the means of standard error estimates (sm_{se}) for non-zero components in Λ are noticeably closer to the corresponding sample standard deviations of estimates (sse) under confirmatory analysis (Web Table 1). Interestingly, the variability of non-zero components in Λ (sse) are comparable between confirmatory (Web Table 1) and exploratory analyses (Web Table 2). This suggests that the restriction of $\lambda_{12} = 0$ imposed in exploratory analysis is adequate to accurately identify the factor structure.

Univariate CACE analysis

Under univariate CACE analysis, covariance pattern models with an unstructured variance-covariance structure are applied to the individual outcome. For individual endpoint, the model for Y^a_{ij}, i^{th} patient's measurement at time point j within compliance type m when assigned to group a, is specified as below:

$$Y^a_{ij}\big|(C_i = m) = \beta_{m0} + \beta_{m1}t_{ij} + \beta_{m2}t^2_{ij} + \beta_{m3}D_i(a)t_{ij} + \epsilon^a_{mij}$$

where $m = c$ or n. Since $D_i(a) = 0$ for never-takers, $\beta_{n3} \equiv 0$. Let $\boldsymbol{\epsilon^a_{mi}} = (\epsilon^a_{mi0}, \epsilon^a_{mi1}, \cdots, \epsilon^a_{miJ})^T$, we assume $\boldsymbol{\epsilon^1_{mi}} \perp\!\!\!\perp \boldsymbol{\epsilon^0_{mi}}$ and $\boldsymbol{\epsilon^a_{mi}} \sim N(0, \Sigma_m)$ where Σ_m has an unstructured variance-covariance structure. Because the variance-covariance matrix in the latent-factor MCACE model is complicated, here covariance pattern models with an unstructured variance-

covariance matrix are employed to avoid model misspecification when conducting univariate CACE analysis.

$$\Sigma_m = \begin{pmatrix} \sigma_{m11} & \sigma_{m12} & \sigma_{m13} & \cdots & \sigma_{m1J} \\ \sigma_{m21} & \sigma_{m22} & \sigma_{m23} & \cdots & \sigma_{m2J} \\ \vdots & \vdots & \vdots & \vdots & \vdots \\ \sigma_{mJ1} & \sigma_{mJ2} & \sigma_{mJ3} & \cdots & \sigma_{mJJ} \end{pmatrix} \tag{S3}$$

In Eqn S3, $\sigma_{mjj'} = \sigma_{mj'j}$ because variance-covariance matrix is symmetric.

We also use the logistic regression to model compliance patterns as shown in Eqn 5. Similar to multivariate analysis, we did not consider covariates and $p_{ci} = p_c = 0.3$.

We assume individuals are followed up at the same fixed time points, which means $t_{ij} = t_j$. The complier average causal effect for individual outcome is

$$E(Y_{ij}^1 - Y_{ij}^0 | C_i = c) = \beta_{c3} t_j.$$

Therefore, the compiler average causal effect under univariate CACE analysis is captured by the coefficient of $D_i(a)t_{ij}$.

A.2 Additional Results in the Application

Web Table 3 reports the estimates and standard errors when all outcomes load on the same latent factor. In this case, we only consider one latent factor ($q = Q = 1$) in Eqn 1 (level-1 model). Besides, β_{1c01} in Eqn 10 (level-2 model) is also set to be 0 because this is a randomized controlled trial. The estimates of parameters in Λ are positive, which implies the potential outcomes Y_{ijk}^a given compliance type m are positively correlated with the latent factor U_{1mij}^a. Because we are interested in the overall CACE captured by the latent factor, the complier average causal effect on the common latent factor at the sixth month

is

$$E(U_{1mi2}^{1}|m=c) - E(U_{1mi2}^{0}|m=c) = \beta_{1c11} * 2 + \beta_{1c21} * 4.$$

The complier average causal effect on the common latent factor is estimated to be 1.447 (SE = 0.636, p-value = 0.023, Web Table 3). Therefore, the latent-factor MCACE model detects an overall significant beneficial CACE of using the AHJ based on the common latent factor.

Web Table 3: Estimates and standard errors for causal treatment effects in the AHJ study

One latent factor only			
Fixed effects estimation†			
Parameter	Estimate	SE	P-value
β_{1c11}	2.818	1.195	0.018
β_{1c21}	-1.047	0.525	0.046
Treatment effect estimation at the sixth month			
	Estimate	SE	P-value
$\text{CACE}_1^{\ddagger}$	1.447	0.636	0.023
Estimation of factor loading matrix Λ			
Parameter	Estimate	SE	
λ_{11}	8.192	0.084	
λ_{21}	3.074	0.571	
λ_{31}	9.302	0.680	
λ_{41}	2.972	0.666	
λ_{51}	8.967	0.737	
λ_{61}	2.872	0.648	

†: Fixed effects estimation section only shows parameters related to treatment effect estimation.

‡: CACE_1 represents overall CACE for six outcomes when considering one latent factor only.

Web Table 4 reports the estimation results of the compliance model when one latent factor is considered. Similar to the situation where two latent factors are considered, the

coefficients for all covariates except high disease activity are negative. Additionally, the coefficients for early disease, high disease activity and older age are statistically significant. Finally, we concluded that the estimation results suggested that younger female patients with longer disease duration and high disease activity were more likely to be compliers.

Furthermore, we also provided the results under the latent factor model with three factors in Web Table 5 and 6.

Web Table 4: Estimation results of compliance model

Covariates	Estimate	Standard error	P-value
One latent factor			
Intercept	-0.799	0.614	0.193
Early disease	-2.362	1.121	0.035
High disease activity	1.365	0.641	0.033
Male	-1.009	0.871	0.246
Older age	-0.956	0.484	0.048

Note: Early disease = 1 if early disease (0-2 years) and = 0 if late disease ($\geq$ 2 years)), high disease activity = 1 if high disease activity (high RAPID4 values) and = 0 if low disease activity (remission, moderate/low RAPID4 values)), Male = 1 if male and = 0 if female, Older age = 1 if above the median age (54.5) and = 0 if otherwise.

Web Table 5: Estimates and standard errors for causal treatment effects in the AHJ study

Three latent factors			
Fixed effects estimation†			
Parameter	Estimate	SE	P-value

β_{1c11}	2.789	1.204	0.021
β_{1c21}	-1.039	0.532	0.051
β_{2c11}	1.986	1.594	0.213
β_{2c21}	-0.884	0.802	0.271
β_{3c11}	1.907	1.175	0.105
β_{3c21}	-0.513	0.581	0.377
	Treatment effect estimation at the sixth month		
	Estimate	SE	P-value
$\text{CACE}_1^{\ddagger}$	1.421	0.620	0.022
$\text{CACE}_2^{\ddagger}$	0.437	0.865	0.613
$\text{CACE}_3^{\ddagger}$	1.761	0.657	0.007
	Estimation of factor loading matrix Λ		
Parameter	Estimate	SE	
λ_{11}	7.836	0.670	
λ_{21}	-		
λ_{31}	8.996	0.679	
λ_{41}	-		
λ_{51}	8.382	0.708	
λ_{61}	-		
λ_{12}	-		
λ_{22}	7.769	0.459	
λ_{32}	-		
λ_{42}	-		
λ_{52}	-		

λ_{62}	-	
λ_{13}	-	
λ_{23}	-	
λ_{33}	-	
λ_{43}	6.528	1.202
λ_{53}	-	
λ_{63}	6.678	1.240

†: Fixed effects estimation section only shows parameters related to treatment effect estimation.

‡: CACE_1 is for the first factor, self-efficacy in disease management; CACE_2 is for the second factor, manage symptoms scale; CACE_3 is for the third factor, interaction with health care providers.

Web Table 6: Estimation results of compliance model

Covariates	Estimate	Standard error	P-value
Three latent factors			
Intercept	-0.843	0.615	0.170
Early disease	-2.399	1.122	0.033
High disease activity	1.451	0.645	0.025
Male	-0.725	0.845	0.391
Older age	-0.973	0.489	0.046

Note: Early disease = 1 if early disease (0-2 years) and = 0 if late disease ($\geq$ 2 years)), high disease activity = 1 if high disease activity (high RAPID4 values) and = 0 if low disease activity (remission, moderate/low RAPID4 values)), Male = 1 if male and = 0 if female, Older age = 1 if above the median age (54.5) and = 0 if otherwise.

Web Table 7: Alternative analysis

Parameter	Estimate	Standard error	P-value
		Under definition D1	
$CACE_1$	1.386	0.521	0.008
$CACE_2$	1.793	0.499	0.0003
		Under definition D2	
$CACE_1$	1.240	0.477	0.009
$CACE_2$	1.671	0.456	0.0002

Note: Definition D1 defines compliers as patients who would use the tool at least three times over six months if assigned to treatment group. The second definition D2 defines compliers as patients who would use the tool at least once over six months if assigned to treatment group. $CACE_1$ is for the first factor, self-efficacy. $CACE_2$ is for the second factor, interaction with health care providers.
